# Supplementary material for: Coronary arterial development is regulated by a Dll4-Jag1-EphrinB2 signaling cascade
Source: eLife. 2019 Dec 4;8:e49977. doi: 10.7554/eLife.49977 (PMC6917494; doi:10.7554/eLife.49977)
Supplement: Transparent reporting form [file elife-49977-transrepform.docx]

***eLife’s* transparent reporting form**

We encourage authors to provide detailed information *within their submission* to facilitate the interpretation and replication of experiments. Authors can upload supporting documentation to indicate the use of appropriate reporting guidelines for health-related research (see [EQUATOR Network](http://www.equator-network.org/%20)), life science research (see the [BioSharing Information Resource](https://biosharing.org/)), or the [ARRIVE guidelines](http://www.plosbiology.org/article/info:doi/10.1371/journal.pbio.1000412) for reporting work involving animal research. Where applicable, authors should refer to any relevant reporting standards documents in this form.

If you have any questions, please consult our Journal Policies and/or contact us: [editorial@elifesciences.org](mailto:editorial@elifesciences.org).

**Sample-size estimation**

- You should state whether an appropriate sample size was computed when the study was being designed
- You should state the statistical method of sample size computation and any required assumptions
- If no explicit power analysis was used, you should describe how you decided what sample (replicate) size (number) to use

Please outline where this information can be found within the submission (e.g., sections or figure legends), or explain why this information doesn’t apply to your submission:

Sample size for each experiment is reported in its corresponding figure legend and in the *Supplementary File 1_Source data*.

A minimum of 3 biological replicates (independent embryos) in each group were considered to detect as significant (alpha=0.05) a between groups difference of 50%

**Replicates**

- You should report how often each experiment was performed
- You should include a definition of biological versus technical replication
- The data obtained should be provided and sufficient information should be provided to indicate the number of independent biological and/or technical replicates
- If you encountered any outliers, you should describe how these were handled
- Criteria for exclusion/inclusion of data should be clearly stated
- High-throughput sequence data should be uploaded before submission, with a private link for reviewers provided (these are available from both GEO and ArrayExpress)

Please outline where this information can be found within the submission (e.g., sections or figure legends), or explain why this information doesn’t apply to your submission:

Experiments were repeated 3 times in a minimum of 3 biological samples.

Except:

For quantifications of IsoB4 (arteries) and Emcn (veins) in E16.5 *Dll4^GOF^;Tie2-Cre* embryos two transgenic embryos were used. For quantifications at E14.5 two controls were used (Figure 4-source data 1, sheet 10).

For quantifications of Dll4 ISH on *Dll4^flox^;Pdgfb-iCreERT2* embryos, two controls and two mutants were used (Figure 3-figure suppl. 1-source data 1, sheet 5).

For quantification of IsoB4 area (arteries) in E16.5 *Mfng^GOF^;Tie2-Cre* embryos, two controls and two transgenic embryos were used (Figure 4-figure supplement 1D,G-source data 1, sheet 11).

RNA-seq data are deposited in the NCBI GEO database under accession number GSE110614 (*Materials and methods*, Page 21)

**Statistical reporting**

- Statistical analysis methods should be described and justified
- Raw data should be presented in figures whenever informative to do so (typically when N per group is less than 10)
- For each experiment, you should identify the statistical tests used, exact values of N, definitions of center, methods of multiple test correction, and dispersion and precision measures (e.g., mean, median, SD, SEM, confidence intervals; and, for the major substantive results, a measure of effect size (e.g., Pearson's r, Cohen's d)
- Report exact p-values wherever possible alongside the summary statistics and 95% confidence intervals. These should be reported for all key questions and not only when the p-value is less than 0.05.

Please outline where this information can be found within the submission (e.g., sections or figure legends), or explain why this information doesn’t apply to your submission:

Canonical Figures:

Page 27, Legend Figure 1 (VM, branching points, EdU+/ERG+)-mean, one-way ANOVA

Page 28, Legend Figure 2 (p27)- mean, one-way ANOVA with Tukey’s multiple comparison tests

Page 29, Legend Figure 3 (IsoB4, Emcn, N1ICD/isoB4, SMA_Notch3) mean, SD, t-test

Page 30, Legend Figure 4 (IsoB4, Emcn, N1ICD/isoB4, SMA_Notch3) mean, SD, t-test

Page 31, Legend Figure 5 (Area Emcn, IsoB4), (N1ICD/isoB4, SMA_Notch3) mean, SD, t-test

Page 32, Legend Figure 6, (Vessel caliber, Distance branching points, Average number of branching points mean, SD, t-test. B-H adjusted p-value.

Page 33, Legend Figure 7 (angiogenesis assays) mean, ANOVA

Figure supplements:

Page 38, Legend Figure 1-figure supplement 4 (CM thickness) mean, one-way ANOVA with Tukey’s multiple comparison tests.

Page 40, Legend Figure 3-figure supplement 1 (Dll4 and Jag1 ISH) mean, SD, t-test

Page 41, Legend Figure 3-figure supplement 2 (SM22a, Notch3) mean, SD, t-test

Page 42, Legend Figure 3-figure supplement 3. (IsoB4, Emcn, N1ICD/isoB4, SMA&Notch3) mean, SD, t-test

Page 43, Legend Figure 3-figure supplement 4 (CM thickness) mean, one-way ANOVA with the Dunnett multiple comparison tests

Page 44, Legend Figure 4-figure supplement 1 (IsoB4, Emcn, N1ICD&isoB4, SMA&Notch3) mean, SD, Student’s *t*-test.

Page 45, Legend Figure 5-figure supplement 1 (CM thickness, BrdU) mean, SD, t-test

Page 46, Legend Figure 7-figure supplement 1 (angiogenesis assays) mean, one-way ANOVA.

Data and statistical analysis: Raw data can be found in *Supplementary File 1_Source data*, an Excel spread sheet containing all the *P*-values, and 95 % confidence intervals for each experiment.

(For large datasets, or papers with a very large number of statistical tests, you may upload a single table file with tests, Ns, etc., with reference to sections in the manuscript.)

**Group allocation**

- Indicate how samples were allocated into experimental groups (in the case of clinical studies, please specify allocation to treatment method); if randomization was used, please also state if restricted randomization was applied
- Indicate if masking was used during group allocation, data collection and/or data analysis

Please outline where this information can be found within the submission (e.g., sections or figure legends), or explain why this information doesn’t apply to your submission:

No clinical studies were included.

**Additional data files (“source data”)**

- We encourage you to upload relevant additional data files, such as numerical data that are represented as a graph in a figure, or as a summary table
- Where provided, these should be in the most useful format, and they can be uploaded as “Source data” files linked to a main figure or table
- Include model definition files including the full list of parameters used
- Include code used for data analysis (e.g., R, MatLab)
- Avoid stating that data files are “available upon request”

Please indicate the figures or tables for which source data files have been provided:

Source data can be found in *Supplementary File 1_Source data*, containing the quantitative data and statistics of (in order of appearance in the MS text):

Sheet 1: Figure 1-figure suppl. 2,

Sheet 2: Fig1,

Sheet 3: Figure 1-figure suppl.4,

Sheet 4: Figure 2,

Sheet 5: Figure 3-figure suppl. 1,

Sheet 6: Figure 3,

Sheet 7: Figure 3-figure suppl. 2,

Sheet 8: Figure 3-figure suppl.3,

Sheet 9: Figure 3-figure suppl. 4,

Sheet 10: Figure 4,

Sheet 11: Figure 4-figure suppl. 1,

Sheet 12: Figure 5,

Sheet 13: Figure 5-figure suppl. 1,

Sheet 14: Figure 6,

Sheet 15: Figure 7,

Sheet 16: Figure 7-figure suppl. 1.
